# Comparative Analysis of Gut Microbiota in Two Populations of Argopecten purpuratus (Lamarck, 1819) Based on 16S rRNA Gene Amplicon Data

**DOI:** 10.1128/MRA.01481-19

**Published:** 2020-03-05

**Authors:** Wilbert Serrano, Raul M. Olaechea, Ulrike I. Tarazona

**Affiliations:** a Molecular Microbiology and Bacterial Genomics Laboratory, Universidad Científica del Sur, Lima, Peru; University of Maryland School of Medicine

## Abstract

Despite the importance of the Peruvian scallop *Argopecten purpuratus* as a major cultivated species, information on its microbiota is limited. Here, we provide a high-throughput sequencing data analysis of 16S rRNA gene amplicons from the distal intestine of *A. purpuratus*. Geographical and seasonal variation of the indigenous gut microbiota is shown.

## ANNOUNCEMENT

The species Argopecten purpuratus (Lamarck, 1819) is the most abundantly harvested and maricultured mollusk in Peru. Scallop aquaculture has become the second most exported product of the Peruvian marine farming industry (1). The natural distribution of this species extends from Paita, Peru (5°S, 81°W), to Bahía Vicente, Chile (37°S, 73°W) (2). Although *A. purpuratus* is not considered an endangered species, its natural stocks are subject to illegal seed-harvesting activities to support the demanding scallop aquaculture industry (3). Building a hatchery of *A. purpuratus* seeds could be a viable solution to this problem; however, the main bottleneck of this nascent activity is the frequent outbreaks of diseases, caused mainly by vibrios (4, 5). The use of probiotics appears to be an eco-friendly alternative for combating bacterial infections in this context; here, we report a high-throughput 16S rRNA gene sequence analysis to study the microbiota in the distal portion of the intestine of *A. purpuratus*.

Twenty adult specimens of *A. purpuratus* were collected by scuba diving in two locations in Peru, Independence Bay (IB; 14.2356S, 76.1920W) and Sechura Bay (SB; 5.65S, 80.96W), in austral spring and autumn (November 2015 and March 2016, respectively). Fecal pellets from 10 exemplars were combined before nucleic acid extraction with a soil DNA extraction kit (Norgen Biotek, Canada).

The V3 and V4 regions of bacterial 16S rRNA genes served as a target for high-throughput sequencing using the default primers 341F (CCTACGGGNGGCWGCAG) and 805R (GACTACHVGGGTATCTAATCC). Library construction and sequencing were done at Macrogen, Inc. (Seoul, South Korea), and sequences were determined using an Illumina MiSeq instrument. The system produced raw data with paired-end reads (301 bp long) as follows: 110,921 (IB, spring), 112,856 (IB, autumn), 117,900 (SB, spring), and 119,909 (SB, autumn). The quality of sequences was evaluated by FastQC package v0.11.8 (6). High-quality sequences were used as input for further 16S microbial diversity analysis following the standard operating procedure (SOP) according to Schloss et al. with the program Mothur v1.39.5 (7) as implemented within the Galaxy platform (www.usegalaxy.org). Briefly, the SOP includes data preparation (demultiplexing/denoising); quality control, which includes chimera filtering, sequence alignment, and clustering; and sequence classification steps. SILVA v128 (8) was used to assign operational taxonomic units (OTUs) at 97% similarity. The VSEARCH tool (9) was used for chimera removal.

**FIG 1:**
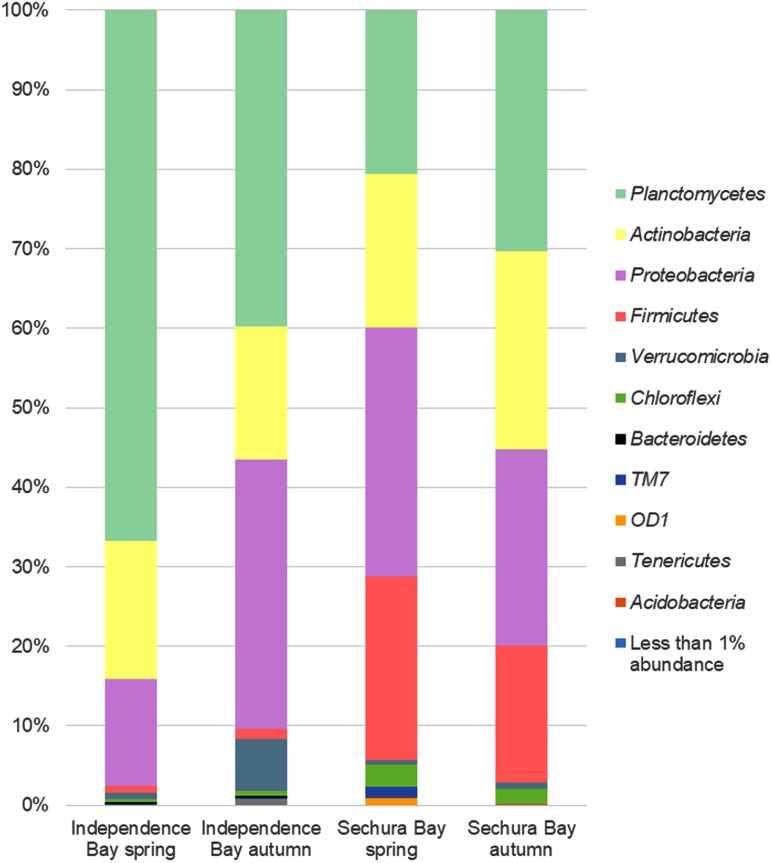
Bar chart representing bacterial diversity of distal segment of intestine of *A. purpuratus* based on 16S rRNA amplicon analysis. The relative abundance (%) at the phylum level is shown.

As can be seen in Fig. 1, the most representative OTUs at phylum level for all examined samples were *Planctomycetes* (21 to 67%), *Actinobacteria* (17 to 25%), and *Proteobacteria* (13 to 34%). This tendency was almost the same regardless of the seasons in which the samples were collected. However, a remarkable feature is that *Firmicutes* were less abundant in IB in both spring and autumn (<2%), while these values were 17 to 23% for SB. Interestingly, *Bacteroidetes* were present only in IB and represented <0.5% of the total abundance. Combined, the abundances of the other phyla were below 1%, except for the candidate division phylum *TM7* (1.4%) for SB in spring and *Chloroflexi* (2 to 3%) for SB in spring and autumn.

This study allowed a comparative analysis of the gut microbial diversity for two populations of the Peruvian scallop *Argopecten purpuratus*.

### Data availability.

The raw 16S rRNA gene amplicon data set was deposited in GenBank under the SRA accession numbers SRX7228172 (IB spring), SRX7228173 (IB autumn), SRX7228174 (SB spring), and SRX7228175 (SB autumn). The associated BioProject accession number is PRJNA380428.
